# IL-2 Regulates SEB Induced Toxic Shock Syndrome in BALB/c Mice

**DOI:** 10.1371/journal.pone.0008473

**Published:** 2009-12-29

**Authors:** Aslam Ali Khan, Shilpee Priya, Bhaskar Saha

**Affiliations:** 1 Washington University in St. Louis, St. Louis, Missouri, United States of America; 2 National Centre for Cell Science, Pune, India; Centre de Recherche Public de la Santé (CRP-Santé), Luxembourg

## Abstract

**Background:**

Toxic Shock Syndrome (TSS) is characterized by fever, rash, hypotension, constitutional symptoms, and multi-organ involvement and is caused by Staphylococcus aureus enterotoxins such as Staphylococcal Enterotoxin B (SEB). SEB binds to the MHC-IIα chain and is recognized by the TCRβ chain of the Vβ8 TCR^+^ T cells. The binding of SEB to Vβ chain results in rapid activation of T cells and production of inflammatory cytokines, such as Interleukin-2 (IL-2), Interferon-γ and Tumor Necrosis Factor-α which mediate TSS. Although IL2 was originally identified as the T cell growth factor and was proposed to contribute to T cell differentiation, its role in TSS remains unexplored.

**Methodology/Principal Findings:**

Mice were injected with D-Gal (25 mg/mouse). One hour after D-Galactosamine (D-Gal) injection each mouse was injected with SEB (20 µg/mouse. Mice were then observed for 72 hrs and death was recorded at different times. We tested Interleukin-12, IFNγ, and IL-2 deficient mice (IL-2^−/−^), but only the IL-2 deficient mice were resistant to SEB induced toxic shock syndrome. More importantly reconstitution of IL-2 in IL-2 deficient mice restored the shock. Interestingly, SEB induced IL-2 production from T cells was dependent on p38MAPK activation in macrophages as inhibition of it in macrophages significantly inhibited IL-2 production from T cells.

**Conclusion:**

This study shows the importance of IL -2 in TSS which has not been previously explored and it also shows that regulating macrophages function can regulate T cells and TSS.

## Introduction

TSS is a superantigen-mediated disease. Superantigens are a group of proteins (*S aureus* toxins in the case of TSS) that are able to activate the immune system by bypassing certain steps in the usual antigen-mediated immune response sequence [1]. Superantigens are not processed within the antigen-presenting cell before being presented to T cells [2], instead, they bind directly to the alpha chain of the major histocompatibility complex, class II (MHC-II), and are recognized by the Vβ chain of T cell receptor to trigger a massive T-cell activation [3]. Staphylococcal Enterotoxin B (SEB), a superantigen, is among the toxins produced by toxigenic strains of *S aureus*, and it can cause TSS in a variety of settings [4]. SEB is the toxin that most commonly causes classic food poisoning. It has also been demonstrated to cause a non-menstrual TSS [5]. In addition, staphylococcal infection in surgical operations can also cause TSS. It is viewed that the mechanism of SEB-induced TSS is a massive T cell activation [6]. While conventional antigens activate only about 0.01% to 0.1% of the T-cell population, super antigens can activate 5%–30% of the entire T cell population [7]. Superantigens lead to a rapid burst of cytokines, especially tumor necrosis factor alpha (TNF-alpha), interleukin-1 (IL-1), and IL-6 and IL-2 [8], [9]. These cytokines are responsible for a capillary leak syndrome and account for many of the clinical signs of TSS [10]. Although TNFα and IFNγ are shown to be the major players in TSS [11], the role of IL-2 is not well studied, despite its original identification as a T cell growth factor and its role in T cell proliferation [12]. Therefore we compared SEB induced TSS in BALB/c and IL-2 deficient (IL-2^−/−^) in BALB/c background mice. Since activation status of antigen presenting cells (APCs) may also play a role in TSS and the crucial role of p38MAPK has already been shown in APCs [13], we studied the role of p38MAPK in SEB induced TSS. Our data demonstrates that IL-2 is essential for SEB induced TSS and that p38MAPK plays an important role in the process.

## Results

### SEB Leads to the Activation of p38MAPK in Peritoneal Macrophages

**Figure 1:**
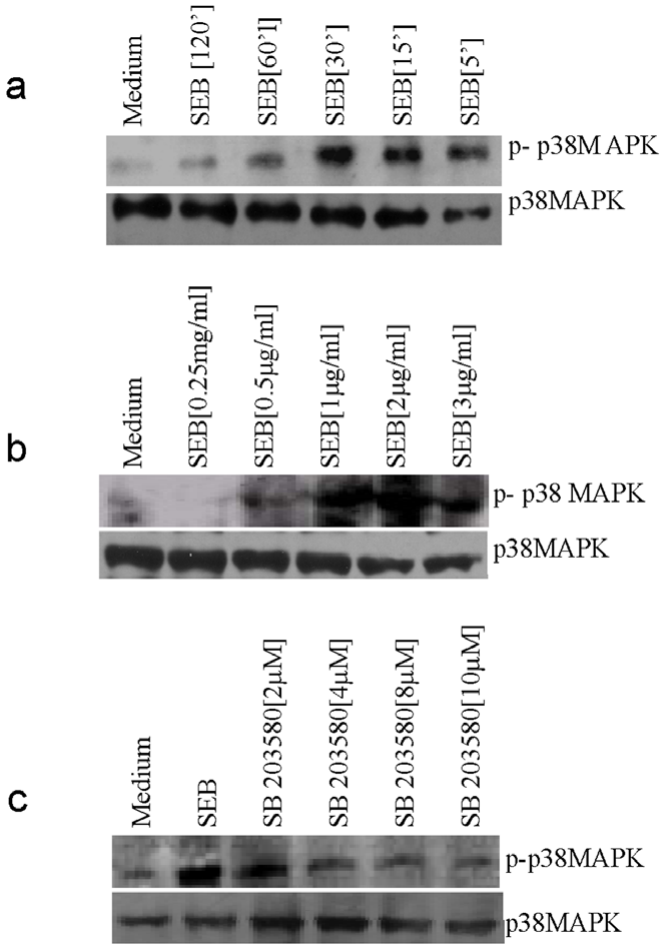
SEB induces p38MAPK phosphorylation in peritoneal Macrophages. (A). PMΦs were stimulated for indicated times and cell lysates were analyzed by western blotting using an anti-phospho p38MAPK specific antibody. Equal loading was confirmed by reprobing the stripped membrane with a p38MAPK specific antibody. (B) Macrophages were stimulated with indicated doses of SEB and cell lysates were analyzed by western blotting using an anti-phospho p38MAPK specific antibody. Equal loading was confirmed by reprobing the stripped membrane with a p38MAPK specific antibody. (C) Macrophages were preincubated with indicated doses of SB-203580 for 2 hrs and stimulated with 2 µg/ml of SEB for 30 minutes, and then the lysates were analyzed by western blotting against phospho p38MAPK. Equal loading was confirmed by reprobing the stripped membrane with a p38MAPK specific antibody.

It was reported recently that ligation of MHC-II by LAG3 or anti-MHC-II leads to activation of p38MAPK [14], [15]. p38MAPK has been shown to induce TNFα in the case of LPS stimulation of macrophages systems. Since TSS is caused by TNFα, therefore we examined the activation of p38MAPK by SEB. Macrophages were treated with SEB for different times and we observed a time dependent increase in p38MAPK activation (Figure 1A) peaking at 30 mins. Similarly, cells were stimulated for 30 mins with indicated doses of SEB and we observed a dose dependent increase in p38MAPK activation peaking at 2 µg/ml (Figure 1B). To determine the effect of SB-203580 (a p38MAPK inhibitor) on the activation of p38MAPK [16], macrophages were incubated with SB-203580 for 2 hrs and then stimulated with SEB (2 µg/ml) for 30 mins; dose dependent inhibition of p38MAPK activation was observed (Figure 1C). This suggests that p38MAPK is activated by SEB in macrophages.

### Inhibition of p38MAPK in Macrophages Modulates T Cell Function

**Figure 2:**
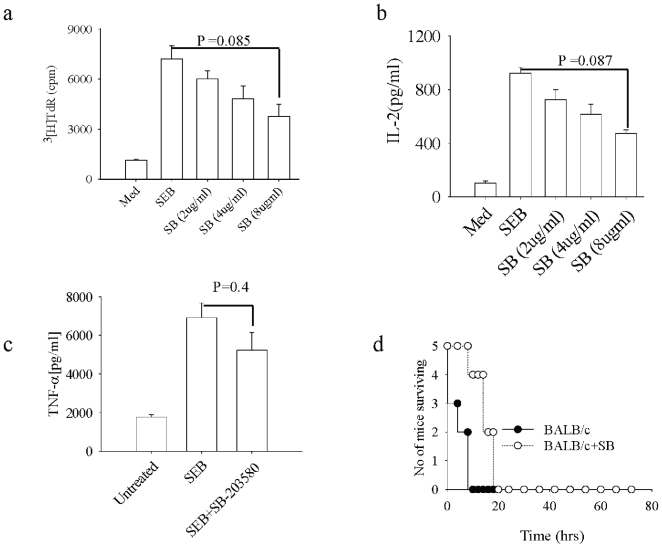
Macrophage pretreatment with SB-203580 inhibits SEB induced IL-2 production in T cells and imparts resistance to SEB induced TSS in BALB/mice. (A) Peritoneal MΦs were preincubated with indicated doses of SB-203580 for 2 hrs and cells were washed and then T cells were added to these macrophage cultures these cultures were then stimulated with SEB (2 µg/ml) for 72 hours and proliferation was measured by adding thymidine at 60 hours for 12 hours. (B) ELISA for IL-2 was done from the supernatant collected at sixty hours from above set. (C) BALB/c mice were injected intraperitoneally (IP) with SB-203580 (25 µg/mouse) and vehicle control (DMSO) (data not shown) one hour before D-Gal injection, then the SEB (20 µg/ml) was injected after one hour after D-Gal injection in the footpad. Mice were bled and ELISA was done to assess serum levels of TNFα (D). Mice were then observed for mortality.

SEB binds to TCR and leads to the proliferation of T cells, and it also signals in APCs [17]. We examined the effect of p38MAPK inhibition in macrophages and its subsequent effect on T cell function. Macrophages were pretreated with SB-203580 (2 µg/ml, 4 µg/ml and 8 µg/ml) for 2 hrs, washed and then were co-cultured with CD4^+^ T cells. SEB (2 µg/ml) was added to the co culture for 72 hrs, and T cell proliferation and IL-2 production were assessed. We observed dose-dependent inhibition of T cell proliferation and IL-2 production (Figure 2A, B). We next tested the effect of SB-203580 on the induction of TSS. Mice were injected with SB-203580 and subsequently challenged with a lethal dose of SEB. Mice pretreated with SB-203580 had lower circulating levels of TNF-α but this was not significant as compared to circulating levels of TNF-α in untreated mice (Figure 2c). SB203580 pretreated mice had prolonged survival as compared to untreated controls (Figure 2d). The observation was consistent with the incomplete protection of mice from TSS.

### IL-2^−/−^ Mice Are Resistant to SEB Induced TSS but IL-12^−/−^ and IFNγ^−/−^ Mice Are Susceptible to TSS

**Figure 3:**
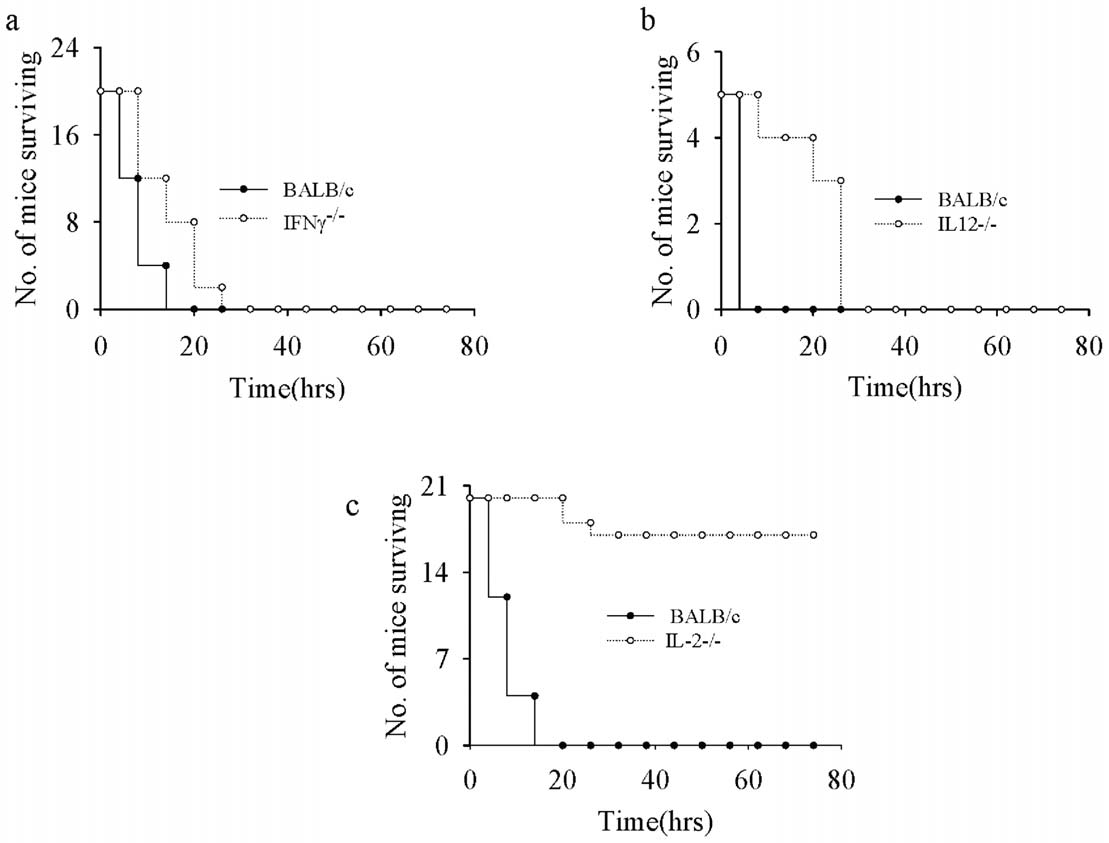
IL-2 knockout mice are resistant to SEB induced TSS. (A) BALB/c and IFNγ^−/−^ mice were injected IP with D-Gal (25 mg/mouse) and after one hour mice were injected with SEB (20 µg/mouse) in the footpad and death was recorded at indicated times. (B) IL-12^−/−^ and BALB/c mice were injected with D-Gal (25 mg/mouse), after one hour mice were injected with SEB (20 µg/ml) and mortality was recorded at indicated times (C) IL-2^−/−^ mice and BALB/c mice were injected in the footpad with SEB (20 µg/mouse) one hour after D-Gal sensitization and mortality was recorded at indicated times.

IFNγ has been implicated as one of the factors in Lipopolysaccharide (LPS) induced shock so we checked whether IFNγ^−/−^ mice are resistant to SEB induced TSS. Mice were challenged with a lethal dose of SEB and mortality was observed. We observed that IFNγ mice were susceptible to SEB induced TSS. Since TSS is the result of high inflammatory cytokine production we checked whether IL-12^−/−^ mice are resistant to SEB induced TSS. WT BALB/c and IL-12^−/−^ mice were challenged with a lethal dose of SEB and observed for death. Much like INFγ^−/−^ mice we found that IL-12^−/−^ mice were susceptible to SEB induced TSS (Fig. 3b), suggesting that IL-12 is not involved in SEB induced TSS. Since SEB induced IL-2 production was inhibited when SB203580-pretreated macrophages were co-cultured with naïve CD4^+^ T cells, we checked TSS induction in IL-2 ^−/−^ mice. Mice were challenged with a lethal dose of SEB and death was recorded at different times. We observed that IL-2^−/−^ mice were absolutely resistant to SEB induced TSS (Fig. 3c) suggesting that IL-2 may be involved in SEB induced TSS.

### IL-2^−/−^ Mice Are Resistant Due to Lower TNFα

**Figure 4:**
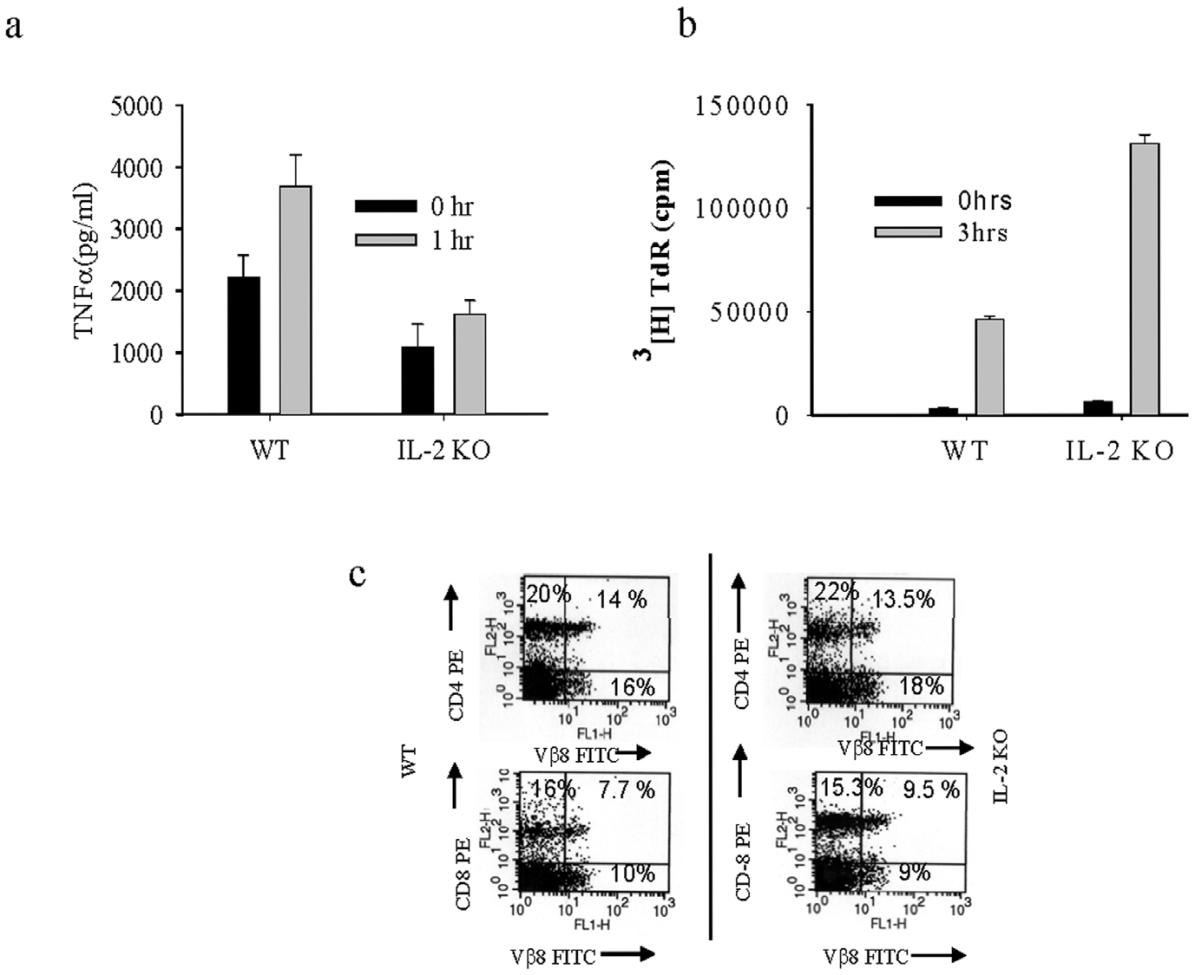
Low level of TNFα in IL-2^−/−^ accounts for the resistance against SEB induced TSS. (A) IL-2^−/−^ and BALB/c mice were bled after one hour after SEB (20 µg/mouse) injection and circulating levels of TNFα were assessed by ELISA. (B) IL-2^−/−^ and BALB/c mice were injected with SEB (20 µg/mouse) in the footpad and spleens were collected after three hours from these mice. Splenocytes were prepared and cultured for 72 hours without further stimulation and proliferation was measured. (C) Spleens were collected from IL-2^−/−^ and BALB/c mice, splenocytes were prepared and FACS was done for CD4 vs. Vβ8 and CD8 vs. Vβ8.

Since IL-2^−/−^ mice are resistant to TSS, we compared production of TNF-α in IL-2^−/−^ and BALB/c wild type mice. SEB-challenged mice were bled after one hour of challenge and ELISA was done to assess circulating levels of TNFα. We found that TNF-α was significantly less in IL-2^−/−^ mice than that observed in BALB/c mice (Figure 4A). We saw a higher basal level of TNF-α in BALB/c and less IL-2^−/−^ mice; this difference could be due to a basal level activity of IL-2 in BALB/c mice. Subsequently, proliferation of splenocytes was tested in wild type and IL-2^−/−^ mice injected with SEB. Proliferation of splenocytes from IL-2^−/−^ mice was found to be higher as previously described [19] (Figure 4B). Since CD4^+^ T cells are responsible for the induction of TSS [20], we wanted to know the status of CD4^+^ cells in IL-2^−/−^ mice. We performed FACS for CD4 vs. Vβ8 and CD8 vs. Vβ8 to check status of CD4^+^ T cells and CD8^+^ T cells from mice injected with SEB for 2 hrs. We found no difference in the population of CD4^+^T cells and CD8^+^ T cells (Figure 4C).

### Reconstitution with rIL-2 Restores the Shock in IL-2^−/−^ Mice

**Figure 5:**
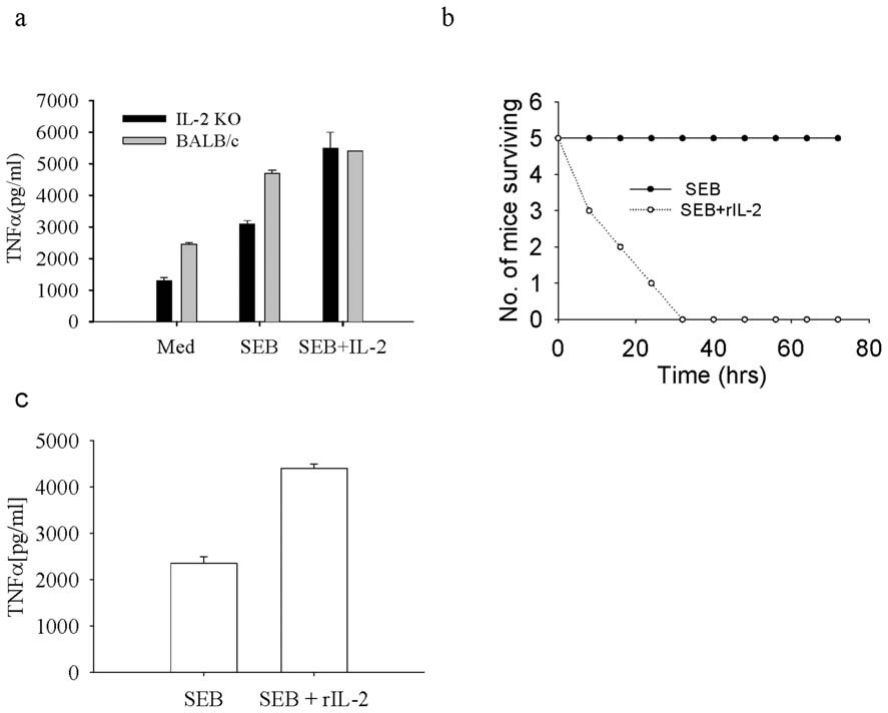
Reconstitution of IL-2 induces shock. (a) Splenocytes were prepared from IL-2^−/−^ and WT mice and stimulated with SEB (2 µg/ml) or SEB + rIL-2 (5 ng/ml) and TNFα levels were measured by ELISA. (b) IL-2^−/−^ mice were injected with SEB (20 µg/mouse), and rIL-2 (500 ng/mouse).Mice were then observed for mortality at indicated times. (c) Above mice were bled one hour after SEB injection and ELISA was done to assess circulating levels of TNF-α in the serum of these mice.

Since IL-2^−/−^ mice were resistant to TSS, we investigated whether reconstitution of IL-2 can restore the TNF-α production. Splenocytes were cultured with or without rIL-2 and were stimulated with SEB. We observed that splenocytes treated with rIL-2 + SEB produced similar levels of TNF-α in both BALB/c and IL-2^−/−^ mice suggesting that rIL-2 can restore TNF-α in the IL-2^−/−^ splenocytes (Figure 5a). To further confirm the role of IL-2 in SEB induced TSS, mice were injected with rIL-2 (500 ng/mouse). Mice were then challenged with a lethal dose of SEB (20 µg/mouse) and death was recorded at different times (Figure 5b). We found that reconstitution of IL-2 restored the shock in IL-2^−/−^ mice confirming the involvement of IL-2 in SEB induced TSS in BALB/c mice. The same mice were bled one hour after SEB injection and ELISA was done for TNF-α. We found an increase in TNF-α level with IL-2 reconstitution, confirming that reconstitution of IL-2 restores TNF-α (Fig. 5c).

## Discussion

SEB binds to MHC-II on APCs and to TCR on T cells producing effects on both cell types. SEB induces IL-12 and TNF-α from APCs and IL-2 and TNF-α from T cells [21]. TNF-α is the common cytokine produced which then induces shock. Our study for the first time explains the importance of signaling molecules like p38MAPK in TSS induction. Since SEB binds to MHC class II and leads to the activation of p38MAPK our results indicate the importance of MHC class II signaling in the regulation of TSS. SEB induces high IL-2 production from T cells. The role of which in the induction of TSS has not been explored. Our results show that IL-2 is involved in induction of TSS, as IL-2^−/−^ mice were found to be absolutely resistant to SEB induced TSS. Interleukin-2 (IL-2) induces high levels of IFN-γ and TNF-α secretion. IL-2 induced secretion of IFN-γ and TNF-α is higher in auto graft recipients than in patients treated by chemotherapy alone. As both TNF-α and IFNγ possess anti-leukemic and anti-infective properties, enhancement of their secretion by IL-2 infusion after autologous bone marrow transplant and induction after chemotherapy is of therapeutic benefit [22]. This study reveals that mice deficient in IL-12 or IFNγ are susceptible to SEB induced TSS whereas the mice deficient in IL-2 are absolutely resistant to SEB induced TSS. CD4^+^ T cells are shown to be involved in mediating the TSS, but the mice deficient in IL-2 have comparable amounts of CD4+ T cells and CD8+ T cells, which proliferate more than WT T cells indicating that there could be some other mechanism of TSS induction. Indeed, it is the IL-2 produced TNF-α which causes TSS as deficiency of IL-2 reduced TNF-α in T cells as well as in the serum of IL-2 deficient mice. Higher proliferation of IL-2 deficient T cells was due to IL-2 induced IL-10 production from T cells, which acts as a regulatory control and regulates T cell proliferation. This phenomenon also helps in maintaining the homeostasis between regulatory T cells and effector T cells. Our study shows that TNF-α plays a key role in the induction of TSS, and complete removal of TNF-α makes the mice more susceptible to diseases like cancer [23]. Our study shows that deficiency of IL-2 reduces the TNF-α production but does not inhibit it completely. This reduction in TNF-α production inhibits TSS in mice and hence could be better a strategy to understand the molecular mechanism of TSS.

## Materials and Methods

All experiments were performed under guidelines and approval of Committee for the Purpose of Control and Supervision of Experiments on Animals (CPCSEA) and Institutional Animal Care of National Centre for Cell Science (NCCS), India and used Committee and Institute approved protocols for the animal experimentation.

### (1) Mice and Other Reagents

SEB, purchased from Sigma (St Louis, MO) was used in all the experiments. Antibodies anti-IL-2, anti-TNF-α, anti-CD4; anti-Vβ8 and anti-CD25 were from BD-PharMingen. Other antibodies used for western blot like anti-pp38MAPK, anti-p38MAPK were from Santa Cruz Biotechnology (Santa Cruz, CA). Thymidine used was from BRITs (Trombay, INDIA). SB-203580 (a p38MAPK inhibitor) was purchased from Calbiochem (La Jolla, CA). IL-2^−/−^, IL-12^−/−^ and IFNγ^−/−^ mice having BALB/c background were purchased from Jackson Laboratories.

### (2) Western Blotting

Western blots were done to determine the p38MAPK phosphorylation. BALB/c mice were injected with 3% thioglycollate intraperitoneally and peritoneal macrophages (PMΦ) were harvested from the peritoneum of the mice and cultured for 12 hrs so that macrophages adhered to the plate. Cells were then washed with RPMI alone (without FCS (Fetal Calf Serum)) to remove the non-adherent cells. Then these cells were stimulated with SEB (2 µg/ml) for different time periods and with different doses of SEB and were also pretreated with a specific inhibitor of p38MAPK in some cases. Cells were lysed with triton X 100 lysis buffer (150 mM NaCl, 25 mM Tris-Cl and 1% triton X 100 pH-7.5) and 20 µg/lane of lysates were loaded onto the gel and blotting was done for p38MAPK phosphorylation and total p38MAPK [15].

### (3) Mortality Assay

Mice used for mortality assay [24] were BALB/c, IL-2^−/−^ (3–4 weeks, IL-12^−/−^ and IFNγ^−/−^ mice (4–6 weeks), weighing 15 g–20 g each. Mice were injected with D-Gal (25 mg/mouse) to sensitize them to SEB induced TSS, as these mice are resistant to TSS without D-Gal sensitization. After 1 hr of D-Gal injection, SEB (20 µg/mouse) was injected in each mouse. Mice were then observed for 72 hrs and death was recorded at different times. For reconstitution & neutralization experiments rIL-2 (R&D systems), (500 ng/mouse) was injected intraperitoneally once only, one hour before D-Gal injection. Control mice injected with saline.

### (4) T Cell Purification

Spleens from BALB/c and IL-2^−/−^ mice were collected and crushed by using one end frosted slide and centrifuged at 1200 rpm for 8 minutes. Cells were treated with Gey's solution to lyse RBCs and then washed with RPMI+10% FCS. Then this cell suspension was loaded onto nylon wool column to get rid of all cells except T cells. Nylon wool column flow through was subjected to CD4+ T cell negative selection using CD4+ T cell isolation kit from Stem Cell Technologies (VC Canada) [25].

### (5) ELISA

The ELISA for the indicated cytokines was performed according to the manufacturers protocol (B.D. PharMingen).

### (6) Serum Preparation

The control and SEB injected mice were bled and sera were prepared after the refraction of the clots and stored at −70°C until used.

### (7) Proliferation

Splenocytes were stimulated with SEB for 72 hours, thymidine was added at the completion of 60 hours of stimulation and counts were taken in Hewlett Packard Radioactive Scintillation counter.

### (8) FACS

Mice were injected with SEB for 2 hrs and then splenocytes were stained for CD4/CD8 vs. Vβ8.
